# Weaning Influences Epithelial Morphology, Gene Expression and Gut Microbiota Composition in Piglets

**DOI:** 10.3390/ani16060961

**Published:** 2026-03-19

**Authors:** Christina Mouchtoglou, Evy Goossens, Marijke Aluwe, Richard Ducatelle, Filip Van Immerseel

**Affiliations:** 1 Livestock Gut Health Team (LiGHT) Ghent, Department of Pathobiology, Pharmacology and Zoological Medicine, Faculty of Veterinary Medicine, Ghent University, 9820 Merelbeke, Belgium; 2 Flanders Research Institute for Agriculture, Fisheries and Food (ILVO), 9090 Merelbeke-Melle, Belgium

**Keywords:** weaning, post-weaning diarrhea, gut health, gut microbiota

## Abstract

Weaning introduces a variety of health-related challenges in piglets, but the relative contributions of the weaning event itself versus biological age at weaning remain unclear. During this period, the gastrointestinal tract has not yet fully developed, adding to the obstacles faced by piglets during this transitory phase in their life, which includes stress from a switch in diet and environment, in addition to potential exposure to pathogens. We investigated the intestinal morphology, expression of genes related to intestinal function and inflammation, and the gut microbiota in 40 piglets weaned at either 3 or 5 weeks of age through complementary analyses: age-matched comparisons (22, 25, 32, 36, and 39 days old) assessed developmental trajectories, while days post-weaning (DPW) comparisons (1 and 4 days post-weaning) isolated acute weaning responses independent of biological age. Animals weaned at 3 weeks of age were divided into five pens of four piglets, while the other group remained with the sow until weaning. At each timepoint, we measured the small intestine length, villus length, crypt depth and mucosal CD3^+^ T-cell infiltration in mid-jejunal tissue. The gene expression of inflammatory markers, tight junction proteins and functional markers was quantified from duodenal and mid-jejunal tissue. The colonic microbiota composition was characterized by 16S rRNA gene sequencing. Both weaning groups showed similar acute morphological responses. However, adaptive gene expression patterns differed significantly. The DPW analysis revealed compensatory mechanisms: at DPW4, the early-weaned piglets exhibited 4-fold higher duodenal IAP than the late-weaned piglets (*p* < 0.001), while the late-weaned piglets maintained higher antimicrobial defenses (IL-8, *p* = 0.031; lysozyme, *p* = 0.027). Additionally, microbiota analysis revealed distinct succession patterns between the two groups. These findings demonstrate that acute physiological responses to weaning are age-independent, but biological maturity fundamentally shapes adaptive mechanisms and recovery trajectories. Early weaning requires compensatory physiological adjustments, while late weaning confers resilience through more stable microbiota and sustained innate defenses.

## Simple Summary

Weaning is a challenging period in a piglet’s life, where the easily digestible sow’s milk is replaced by solid feed, in addition to environmental stressors such as new litter mates and housing conditions. In this study, we evaluate the effect of different weaning ages on the piglet’s health. More specifically, we investigate whether piglets weaned at different ages show differences in morphological and inflammation-related parameters and gut microbiota. This study helps to shed light on the effects weaning has on gastrointestinal health and on potential avenues to focus on in order to support piglet growth and health.

## 1. Introduction

A piglet’s life is marked by numerous stressors, with weaning being one of the most significant. Weaning is the stage in which young piglets begin to transition from consuming its mother’s milk to solid feed. Under natural conditions, this process occurs gradually when piglets are around 12 to 14 weeks old. However, in industrial settings, piglets are typically weaned much earlier, at just 3 to 4 weeks of age, introducing additional stress during a critical stage of development.

Early weaning allows for more piglets weaned per sow per year. However, this intensive system has adverse effects on the young piglet’s health. More specifically, a switch to less digestible feed can contribute to a sudden shift in microbiota composition [1]. Furthermore, impaired growth during the weaning stage can be attributed to the destruction of intestinal villi, which play an important role in nutrient absorption. Weaning is known to cause villus atrophy and crypt hyperplasia by disrupting the balance of proliferation and apoptosis of the intestinal cells. Additionally, an increase in intestinal permeability and expression of pro-inflammatory cytokines, accompanied by gut microbiome changes [1,2,3], ultimately affects nutrient absorption. Early weaning, alongside genetics, environmental conditions and infection with enterotoxigenic *Escherichia coli* (ETEC), are all considered predisposing factors for post-weaning diarrhea (PWD). This is an enteric disease that impacts the growth and welfare of the animals by causing them to have watery discharge, weight loss and dehydration [4,5,6]. While data are available that compare pre- and post-weaning characteristics of the intestinal environment, studies comparing weaned versus non-weaned animals at identical ages are scarce.

Weaning introduces a variety of challenges that affect gut homeostasis, which relies on the interaction between multiple factors such as the gut microbiota, immune system and gut barrier function. More specifically, cytokines like IL-8, TGF-β and IFN-γ involved in inflammatory responses and regulatory actions, such as barrier function and homeostasis of the intestinal epithelium, could be affected by weaning [7,8]. Zonula occludens (ZO-1) and occludin are tight junction proteins that play an important role in epithelial barrier function; therefore, their disruption leads to complications such as a “leaky gut”, increasing the risk of nutrient loss and pathogenic bacteria entering the circulation [9].

In addition, porcine beta-defensin-1 (pBD-1), lysozyme and intestinal alkaline phosphatase (IAP)—produced by intestinal epithelial cells and having antibacterial properties that target the bacterial cell wall, and thus, are part of the innate immune system—could also be influenced by weaning. Beta-defensins are small cationic proteins that penetrate bacterial cells walls [10]. Meanwhile, lysozyme targets the peptidoglycan layer of the bacterial cell wall, along with regulating innate immune responses [11]. IAP is a small intestinal brush-border enzyme that dephosphorylates (and detoxifies) LPS [12] and extracellular ATP, with the latter resulting in an increase of bicarbonate ions that regulate duodenal pH [13,14], hence affecting digestion.

Glucagon-like peptide 2 (GLP-2) is a hormone secreted by endocrine cells called L-cells, which are predominantly found in the porcine colon and ileum, with their density decreasing further up the gastrointestinal tract (GIT) [15]. GLP-2 is co-encoded with GLP-1 from the proglucagon gene. This precursor protein undergoes posttranslational processing in order to give rise to GLP-2, which responds to nutrients [16,17] and whose prime functions include energy absorption, digestion, maintaining mucosal morphology and intestinal integrity [18,19]; it also stimulates intestinal growth and function when damaged or injured [17]. As entero-endocrine cells are regulated by luminal signals, production of hormones secreted by L-cells, such as GLP-2, could be affected by weaning as well.

As weaning significantly disrupts the delicate balance between the host physiology and the gut microbiota, understanding these mechanisms is critical for developing and improving strategies to mitigate early-weaning-associated challenges. However, distinguishing the effects of weaning age from the weaning event itself presents a methodological challenge. Age-matched experimental designs (comparing piglets at the same biological age) necessarily compare weaned animals to still-nursing animals, conflating the acute effects of weaning with age-dependent developmental differences. Conversely, time-post-weaning designs (comparing piglets at the same interval after weaning) introduce differences in biological maturity between groups, making it impossible to isolate the effect of the developmental stage at weaning from biological age per se. We addressed this by using two complementary analyses. First, we compared both groups at the same biological ages (22, 25, 32, 36, and 39 days old) to understand how weaning age affects the overall development over time. Second, we compared both groups at the same days after weaning (1 and 4 days post-weaning) to see how biological maturity at time of weaning changes how piglets respond to the weaning stress itself.

Therefore, in the current study, we aimed to determine (1) how weaning at 3 versus 5 weeks of age affects the intestinal host response and colonic microbiota composition across matched biological ages, and (2) whether biological maturity at weaning influences the acute adaptive response to weaning stress at matched days post-weaning.

## 2. Materials and Methods

### 2.1. Experimental Design and Sample Collection

The study was conducted in accordance with the guidelines of the Ethical Committee of the Research Institute for Agriculture, Fisheries and Food (ILVO) (Merelbeke-Melle, Belgium), under authorization number 2017:308, in accordance with the EU Directive 2010/63/EU. Ethical review and approval were waived for this study, as according to Belgian and EU legislation (Council Directive 86/609/EEG), no procedures requiring approval from the local ethics committee were carried out.

A total of 40 piglets were used. These animals were the offspring of RA-SE hybrid sows crossed with a Piétrain boar, and were supplied by the Institute for Agricultural, Fisheries and Food Research (ILVO) (Merelbeke-Melle, Belgium), where the experiment was carried out on site. Four sows of comparable parity and productivity were selected, and 10 piglets per sow were enrolled in the study with an average weight of 5.5 kg ± 0.71 kg, weighed at 19 days of age. Within each litter, 5 random piglets were weaned at 21 days of age (3 weeks), and the remaining 5 at the age of 35 days (5 weeks). After weaning, piglets were housed in groups of four, with each group comprising one piglet from each sow to ensure balanced representation. After weaning, animals had *ad libitum* access to feed throughout the study (Table 1).

Sampling was performed at five time points: 22, 25, 32, 36, and 39 days of age. At each time point, two piglets per sow—one piglet weaned at 3 weeks and one suckling piglet/weaned at 5 weeks—were selected, yielding a total of eight piglets per sampling day (four from each weaning group). This design allowed for two complementary comparisons: (i) the effect of weaning *per se* by comparing weaned and age-matched suckling piglets at identical ages (days 22, 25 and 32), and (ii) the effect of weaning age by comparing piglets weaned at either 3 or 5 weeks at both 1 and 4 days post-weaning. All piglets were anesthetized intramuscularly with a mixture of 0.15 mL/kg of zolazepam and tiletamine (Zoletil100^®^, Virbac, Leuven, Belgium) and xylazine (Xyl-M^®^ 2%, VMD, Arendonk, Belgium; 0.15 mL/kg) before being euthanized with 200 mg/mL Pentobarbital Sodium (Medini NV, Oostkamp, Belgium).

Piglets were weighed at the time of sampling, and the length of the small intestine was recorded during the necropsy. Colonic contents were collected for 16S rRNA sequencing, immediately snap-frozen in liquid nitrogen, and stored on dry ice until transferred to −70 °C for long-term storage. Mid-jejunal tissues were fixed in 4% phosphate-buffered formaldehyde for histological evaluation. An additional duodenal and mid-jejunal tissue samples were rinsed in sterile phosphate-buffered saline (PBS) and stored on dry ice prior to transfer to −70 °C for gene expression analysis.

### 2.2. Intestinal Morphology and Measurements

Mid-jejunal tissue segments were fixed in 4% phosphate-buffered formaldehyde for 24 h before being embedded in paraffin wax and sectioned at 5 μm. The sections were automatically deparaffinized in xylene and rehydrated in isopropylene, 95% ethanol, and 50% ethanol (Shandon Varistain-Gemini, Thermo Fisher Scientific, Carlsbad, CA, USA) and stained with hematoxylin and eosin. The villus length and crypt depth were assessed using a PC-based image analysis system (Leica Application Suite V4, LAS V4; Leica, Diegem, Belgium). The villus length was measured from the tip of the villus to the crypt–villus junction. The crypt depth was measured from its base up to the crypt–villus invagination. Measurements were taken from at least 7 randomly selected villi/crypts followed by calculating the average per animal. The length of the small intestine (from duodenum to ileum) was measured for each piglet at the time of dissection. The length is expressed relative to each piglet’s live bodyweight.

### 2.3. CD3 Immunohistochemistry

The mid-jejunal sections were automatically deparaffinized in xylene and rehydrated in isopropylene, 95% ethanol, and 50% ethanol (Shandon Varistain-Gemini, Thermo Fisher Scientific, Carlsbad, CA, USA), followed by antigen retrieval with a pressure cooker in citrate buffer (10 mM, pH 6). The endogenous peroxidase activity was blocked by applying peroxidase blocking reagent (Agilent Ref.S202386-2, Diegem, Belgium) for 5 min. To detect CD3^+^ T-cells, mid-jejunal sections were labeled with polyclonal rabbit anti-human CD3 (A0452, Dako, Diegem, Belgium) at a 1/100 dilution for 30 min at room temperature, followed by incubation with labeled polymer–horseradish peroxidase (HRP) anti-rabbit IgG (Envision + System-HRP [DAB; Agilent Ref.K400311-2]) for 30 min. Finally, the slides were counterstained with hematoxylin before dehydration and mounting. The CD3^+^ T-cells area percentage in the mid-jejunum was quantified using 4 representative fields of view per section (×10 objective) and using the LAS V4.1 program for the analysis.

### 2.4. RNA Extraction from Tissue and cDNA Synthesis

RNA from 30–40 mg tissue of duodenum or mid-jejunum was extracted using the Aurum Total RNA Mini Kit (Bio-Rad, Lokeren, Belgium), following the manufacturers’ instructions. An additional DNase treatment was performed to remove any remaining gDNA using the TURBO DNA-free™ Kit (Thermo Scientific, Merelbeke-Melle, Belgium), and the gDNA was stored at −70 °C.

cDNA synthesis was performed using the iScript cDNA Synthesis Kit (Bio-Rad, Lokeren, Belgium) and 1 µg RNA template. The resulting cDNA was diluted 1:5 in nuclease-free water and stored at −20 °C until further use.

### 2.5. qPCR for Relative Quantification of Tight Junction Proteins, Pro-Inflammatory Cytokines, Lysozyme, GLP-2, and Intestinal Alkaline Phosphatase in Duodenum and Mid-Jejunum

To identify stable reference genes for normalization, the expression stability of 5 housekeeping genes (HKGs)—RPL19, RPL4, B2M, cyclophilin, and GAPDH—on both the duodenum and mid-jejunum segments was checked. Primer sequences are listed in Table 2. The gene stability was evaluated using the GeNorm M and coefficient of variance (CV) values, calculated with qbase+ (Biogazelle, Gent, Belgium). In the duodenum section, the genes B2M, cyclophilin and GAPDH were identified as the most stable reference genes. For the mid-jejunum, the most stable genes were B2M, cyclophilin, RPL19 and RPL4.

The relative expressions of IL-8, IFN-γ, TGF-β, pBD1, IAP, GLP-2, lysozyme, ZO-1 and occludin were quantified in both the duodenum and mid-jejunum. The qPCR was run in a 384-well plate containing technical triplicates for each sample using the CFX OPUS 384 system (Bio-Rad). Each 12 μL reaction contained 2 μL cDNA, 6 μL of SensiMix™ SYBR^®^ No-ROX (GC Biotech, Waddinxveen, The Netherlands), 0.5 μM of each primer (except for IFN-γ, which required 0.7 μM), and the remaining volume was HLPLC-grade water. The qPCR thermal cycling conditions were as follows: initial denaturation at 95 °C for 10 min, followed by 40 cycles of 95 °C for 30 s, 58.1 °C for 30 s, and 72 °C for 30 s. A melt curve analysis was performed post-amplification by increasing the temperature from 60 °C to 95 °C in 0.5 °C increments every 5 s to confirm the specificity of amplification.

### 2.6. DNA Extraction from Intestinal Content and 16S rRNA Gene Sequencing

DNA was extracted from 100 mg colon content using the cetyltrimethylammonium bromide (CTAB) method, as previously described [28]. The quality and concentration of the DNA were examined spectrophotometrically (NanoDrop; Thermo Scientific, Waltham, MA, USA).

To characterize the colonic microbiota taxonomically, the V3 to V4 hypervariable region of 16S rRNA gene was amplified using the primers S-D-Bact-0341-b-S-17 (5′-CCTACGGGNGGCWGCAG-3′) and S-D-Bact-0785-a-A-21 (5′-GACTACHVGGGTATCTAATCC-3′) [29], and PCR amplification was performed as described by Aguirre et al. 2019 [28]. The DNA concentration of the resulting libraries was quantified using a Quantus Fluorometer (Promega, Madison, WI, USA). Final libraries were pooled in equimolar concentrations (10 nM) and sequenced using Illumina MiSeq v3 technology (2 × 300 bp, paired-end) at Macrogen (Amsterdam, The Netherlands).

Demultiplexing of the amplicon dataset and deletion of the barcodes were done by the sequencing provider. All further processing was performed in R (v4.2.1). Raw sequence reads were trimmed, quality-filtered and dereplicated using the DADA2 algorithm (v1.24.0). An initial amplicon sequence variant (ASV) table was constructed before chimeras were identified using the *removeBimeraDenovo* function. Finally, taxonomy was assigned using DADA2’s native naïve Bayesian classifier against the Silva database (v1.38). To construct a phylogenetic tree, multiple sequence alignment was performed using the DECIPHER (v2.24.0) algorithm, after which a neighbor-joining tree was constructed using *phangorn* (v2.10.0). This neighbor-joining tree was used as the starting point to fit the final GTR + G + I (Generalized time-reversible with Gamma rate variation) maximum likelihood tree. The resulting phylogenetic tree and ASV table were loaded into *phyloseq* (v1.42.0), after which potential contaminant chloroplastic and mitochondrial ASVs were removed from the data set.

### 2.7. Statistics

Statistical comparisons of animal weight and small intestinal length between groups were performed using the Mann–Whitney U test in GraphPad Prism (version 8.4.3; GraphPad Software, San Diego, CA, USA). All other statistical analyses were performed in R (v4.2.1). Linear mixed-effects models were used to assess the effects of piglet age (*fAge_days*), weaning age group (*Group*), and their interaction on various response variables, including gene expression levels and microbial alpha diversity indices (Chao1 richness or Shannon diversity). The model was

Response ~ *fAge_days* × *Group* + (1 + *fAge_days* | *SowID*)

where *fAge_days* (factorized age) and *Group* were included as fixed effects, along with their interaction. Random intercepts and random slopes for *fAge_days* were included for each sow (*SowID*) to account for repeated measurements and sow-level variability. Models were fitted using the *lme4* and *lmerTest* package in R. When significant interactions were detected, estimated marginal means (EMMs) were computed using the *emmeans* package. *p*-values were adjusted for multiple comparisons using Tukey’s method.

To explicitly disentangle the effect of the weaning event from biological age, two complementary analyses were conducted. The primary analysis compared piglets at matched biological ages (22, 25, 32, 36, and 39 days old) to assess the combined influence of age and weaning status. An additional analysis was conducted focusing specifically on 1 and 4 days post-weaning (DPW) (respectively, days 22 and 25 for piglets weaned at 3 weeks, and day 36 and 39 for piglets weaned at 5 weeks). This DPW analysis allowed us to assess whether biological maturity at weaning influences the acute physiological response to weaning stress, independent of biological age. The results of this complementary analysis are presented in Supplementary Materials S2.

The beta diversity was calculated using the Bray–Curtis dissimilarity metric. The homogeneity of group dispersions (i.e., variance within groups) was assessed using the *betadisper* function, as it is a prerequisite for interpreting PERMANOVA results. The effects of weaning age group (*Group*), sampling day (*fAge_days*), and their interaction on microbial community structure was evaluated using PERMANOVA (adonis2 function), including *SowID* as a covariate. Post hoc pairwise comparisons for PERMANOVA were performed using the *RVaideMemoire* package (pairwise.perm.manova function) with FDR-adjusted *p*-values. Because the assumption of equal dispersion was violated for at least one comparison (day 39), PERMANOVA results were validated using the Wd* test [30], which does not require equal group variance.

Differential abundance analysis at the family and genus levels was conducted using LinDA from the *MicrobiomeStat* package.

## 3. Results

### 3.1. Early-Weaned Piglets Weigh Less but Have a Longer Small Intestine

**Figure 1:**
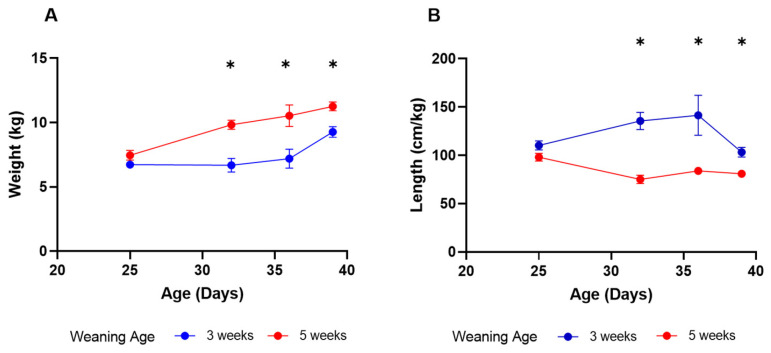
(**A**) Weights of piglets weaned at 3 and 5 weeks of age. (**B**) Lengths of small intestine-to-body weight ratios of piglets from 3- and 5-week weaned groups. Weaning at 3 weeks of age resulted in a higher intestinal length per body weight (kg) as compared with piglets that were not yet weaned (D32) or piglets weaned at 5 weeks of age (D36 and D39). Asterisks above timepoints represent the following level of significance: “*” 0.05.

For this study, we first evaluated the piglet’s weight and small intestinal length. The animals were weighed at D25, D32, D36 and D39 (Figure 1). On D25, the weights of the piglets did not differ (*p* = 0.171). On D32, however, suckling piglets weighed significantly more than those weaned at 3 weeks of age. This difference remained significant for the rest of the sampling days (D32, *p* = 0.029; D36, *p* = 0.029; D39, *p* = 0.029).

Furthermore, we measured the length of the small intestine, corrected for body weight, for each time point (Figure 1). At D32, there was a significantly higher small intestinal length in the weaned piglets as compared with the suckling piglets of the same age (*p* = 0.029). This difference remained significant when both groups of piglets were weaned (weaning at 3 weeks vs. 5 weeks; D36, *p* = 0.029; D39, *p* = 0.029).

### 3.2. Weaning Significantly Increases Crypt Depth and Influx of CD3^+^ T-Cells in the Mid-Jejunum

**Figure 2:**
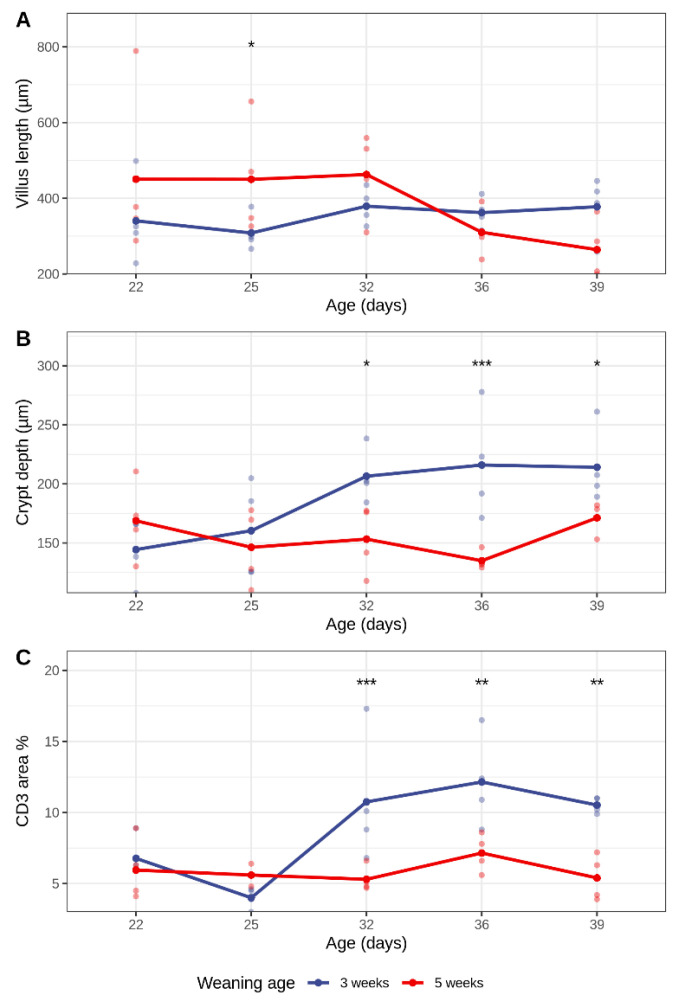
(**A**) Villus length, (**B**) crypt depth and (**C**) CD3^+^ T-cell area percentage of the mid-jejunum during each of the five sampling timepoints in piglets from each group (n = 4). Asterisks above timepoints represent the following levels of significance: “***” 0.001, “**” 0.01, “*” 0.05.

The villus length was not affected by the age of the animals. Also, no major effects of weaning or weaning age on the villus length in the mid-jejunum were observed, with the only exception on D25 (i.e., 4 days after weaning), where the weaned animals had shorter villi than the suckling piglets of the same age (group weaned at 5 weeks) (*p* = 0.046, Table S2, Figure 2A, Supplementary Materials S2).

Weaning caused a significant increase in crypt depth in the mid-jejunum, which was observed as early as 10 days post-weaning (3 weeks group: D22–D32: *p* = 0.052), and reached significance at 15 days post-weaning (3 weeks group: D22–D36: *p* = 0.038, D22–D39: *p* = 0.05). In contrast, no significant difference in crypt depth across ages was observed in the suckling piglets, suggesting a more stable crypt depth over time. When comparing groups at each age, the crypt depth was significantly greater in piglets weaned at 3 weeks compared with those weaned at 5 weeks at D32 (*p* = 0.011), D36 (*p* = 0.0005), and D39 (*p* = 0.035). The difference at D32 reflects the immediate impact of weaning (weaned vs. suckling), whereas at D36 and D39, both groups were weaned, showing the persistence of this effect. This indicates that weaning and not biological age was associated with deeper crypts.

Early weaning resulted in a significant increase of CD3^+^ T-cell area percentage on D32 (*p* = 0.032), D36 (*p* = 0.009) and D39 (*p* = 0.010) compared with D25, suggesting a prolonged inflamed status. Suckling piglets showed no differences across ages. When evaluating between-group differences at each age, the CD3^+^ T-cell area percentage was significantly higher in piglets weaned at 3 weeks versus 5 weeks at D32 (*p* = 0.001), D36 (*p* = 0.002) and D39 (*p* = 0.001). At D32, this comparison reflects weaned (3-week group) versus age-matched suckling piglets (5-week group), suggesting an immediate effect of the weaning event on T-cell infiltration. Importantly, the difference persisted at D36 and D39, when both groups were weaned, indicating a sustained effect associated with the earlier weaning age. To determine whether these morphological responses were driven by the weaning event itself or by the biological age at weaning, we compared piglets at matched days post-weaning (DPW). This analysis revealed no significant effect on the crypt depth or villus length, but days post-weaning was independently associated with an increased CD3^+^ area (*p* = 0.005), indicating that the weaning process itself contributed to the expansion of intestinal T-cell populations, regardless of weaning age (Supplementary Materials S2).

### 3.3. Relative Expression of Genes of Interest in Duodenum and Jejunum

To assess the impact of weaning age on mucosal inflammation, antimicrobial defense, and barrier function, expression levels of genes encoding IFN-γ, IL8, TGF-β, pBD1, IAP, GLP-2, lysozyme, ZO-1, and occludin were quantified in the duodenum and mid-jejunum. The gene expression was compared across five timepoints within each weaning group and between the two groups at each timepoint. To further isolate the effects of the weaning from weaning age, an additional analysis was performed, focusing specifically on 1 and 4 days post-weaning (DPW) (Supplementary Materials S2).

**Figure 3:**
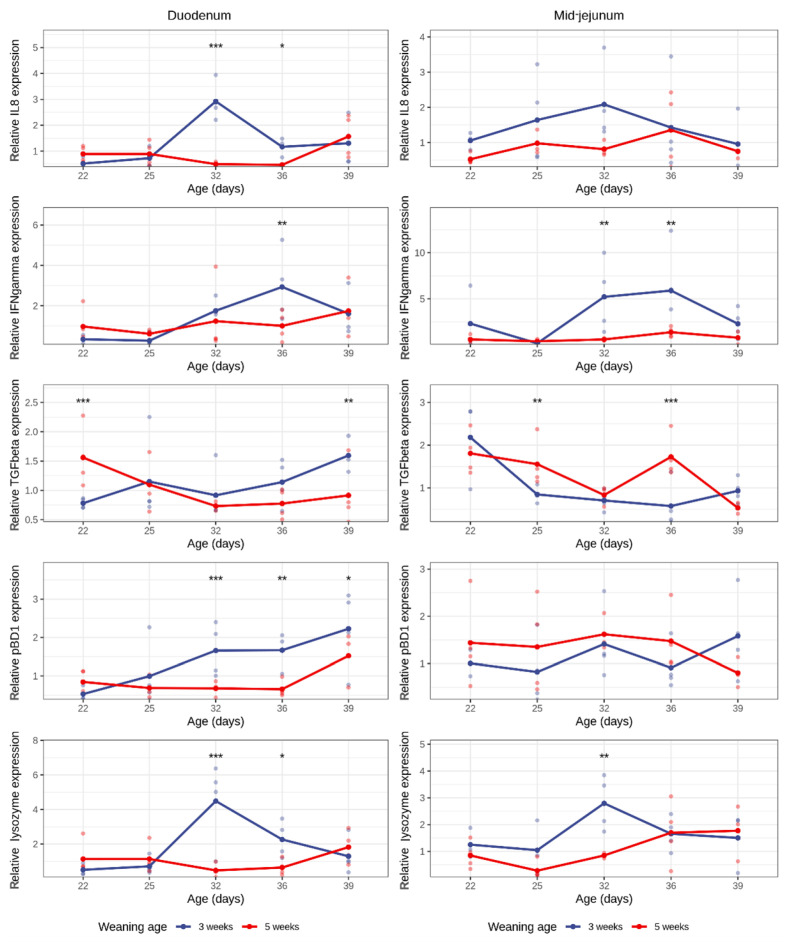
Duodenal and mid-jejunal relative expressions of IL-8, IFN-γ, TGF-β, pBD1, and lysozyme in the 3- and 5-week weaning age groups during the 5 sampling timepoints in piglets from each group (n = 4). Asterisks above timepoints represent the following levels of significance: “***” 0.001, “**” 0.01, “*” 0.05.

Weaning significantly altered the IL-8 and lysozyme expressions in the duodenum, leading to a peak in gene expression at 32 days of age (i.e., 11 days post-weaning). This increase in IL-8 and lysozyme expression was not due age of the animals, as no effect of age was observed in the suckling piglets (Figure 3, Table S5). When the expression was analyzed relative to days post-weaning, both IL-8 and lysozyme levels were confirmed to rise in response to weaning itself, highlighting a weaning-driven effect (Supplementary Materials S2). In the mid-jejunum, the weaning effect was less pronounced, resulting in a more subtle increase in lysozyme at 11 days post-weaning. No effect of either the age or weaning on IL-8 expression in the mid-jejunum was observed.

Within both the 3-week and 5-week weaned groups, no statistically significant differences in the IFN-γ expression were observed between time points in both the duodenum and mid-jejunum. However, in the early-weaned group, the IFN-γ expression at D36 tended to be higher compared with D25 (duodenum: *p* = 0.053, mid-jejunum: *p* = 0.076), suggesting a possible transient increase followed by a decline. Between-group comparisons revealed a significantly higher IFN-γ expression in early-weaned piglets at D32 (mid-jejunum: *p* = 0.004) and D36 (duodenum: *p* = 0.003, mid-jejunum: *p* = 0.004). No significant differences were observed at other timepoints.

In the duodenum of suckling piglets (5-week group), the TGF-β expression decreased with the age of the animals (D22–D32: *p* = 0.017, D22–D36: *p* = 0.060). This could not be observed in the duodenum of weaned piglets (3 weeks group), where no difference between the different timepoints could be observed. At the start of the experiment, the TGF-β expression in the duodenum of suckling piglets was significantly higher than in the one-day weaned piglets (D22, *p* = 0.001). This is in contrast to the last timepoint, where the duodenal TGF-β expression was increased in early-weaned piglets (3-week group) as compared with the piglets that were only 4 days weaned (5-week group) (D39, *p* = 0.002). However, in the mid-jejunum, there appears to be a clear weaning effect with a reduced TGF-β expression after weaning (3 weeks group, D22–D32: *p* = 0.029, D22–D36: *p* = 0.006), leading to a significant lower TGF-β expression in the early-weaned piglets as compared with age-matched suckling piglets (5 weeks group) at D25 (*p* = 0.008) and D36 (*p* = 0.0002). This weaning-driven decrease was further supported by the DPW analysis, which showed a significant effect of days post-weaning on the mid-jejunal TGF-β expression (*p* = 0.003), confirming that the reduction in expression is associated with the weaning event rather than biological age of the piglets (Supplementary Materials S2).

In the duodenum, pBD1 expression increased progressively after weaning in the early-weaned group, reaching significance at D39 compared with D22 (*p* = 0.041). In contrast, no significant age-dependent changes in pBD1 expression were observed within the suckling piglets weaned at 5 weeks of age. Moreover, pBD1 expression was significantly higher in piglets weaned at 3 weeks compared with suckling piglets at D32 (i.e., 11 days post-weaning vs. suckling piglets: *p* = 0.001), D36 (i.e., 15 days post-weaning vs. 1 day post-weaning: *p* = 0.002), and D39 (18 days post-weaning vs. 4 days post-weaning: *p* = 0.023) of age. No significant differences were observed at 22 or 25 days (*p* > 0.16). There were no significant changes in the pBD1 expression in the mid-jejunum over time and between groups.

**Figure 4:**
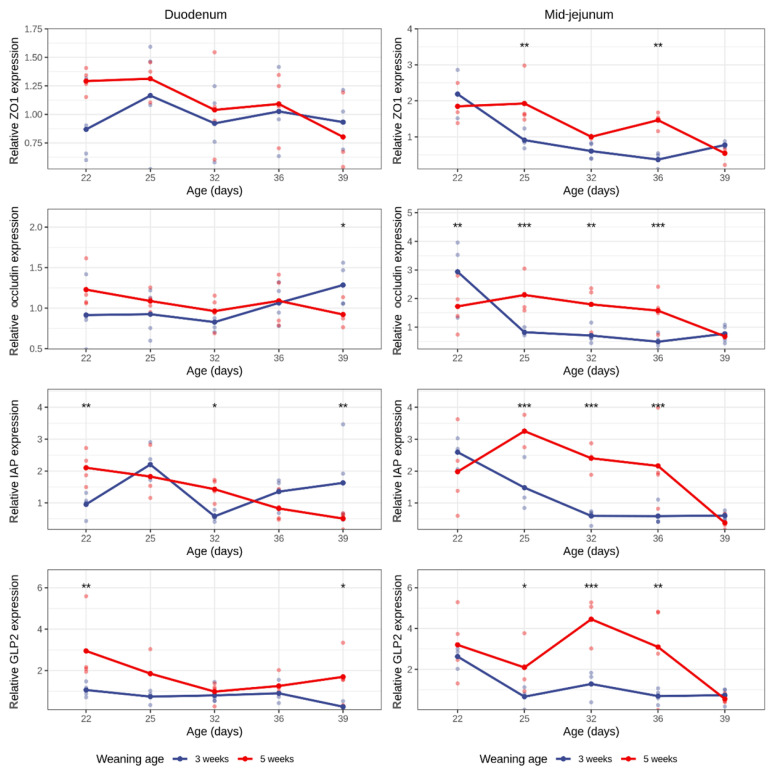
Duodenal and mid-jejunal relative expressions of ZO-1, occludin, IAP and GLP-2 in the 3- and 5-week groups during the 5 sampling timepoints in piglets from each group (n = 4). Asterisks above timepoints represent the following levels of significance: “***” 0.001, “**” 0.01, “*” 0.05.

The gene expressions of the tight junction proteins ZO-1 and occludin in the duodenum was not affected by weaning or age, with the only exception on D39, in which the occludin expression was higher in piglets weaned at 3 weeks of age (i.e., 18 days post-weaning vs. 4 days post-weaning: *p* = 0.041) (Figure 4). This difference was not reflected in the DPW analysis, indicating that it may not represent a consistent early post-weaning effect. In the mid-jejunum, no age-related changes in ZO-1 and occludin expression could be observed in the 5-week weaned group. In contrast, early-weaned piglets showed a strong downward trend in ZO-1 and occludin expression in the mid-jejunum after weaning (ZO-1 expression in the 3-week group, D22 vs. D32: *p* = 0.049, D22 vs. D36: *p* = 0.022, D22 vs. D39: *p* = 0.082) (occluding expression in the 3-week group, D22 vs. D32: *p* = 0.096, D22 vs. D36: *p* = 0.031, D22 vs. D39: *p* = 0.159). Furthermore, when looking into between-group differences, piglets in the 5-week weaned group had higher expressions of ZO-1 and occludin compared with the early-weaned piglets (ZO-1 expression, D25: *p* = 0.002, D36: *p* = 0.001) (occludin expression, D22: *p* = 0.009, D25: *p* = 0.0002, D32: *p* = 0.004, D36: *p* = 0.0008). These findings were supported by the DPW analysis, which showed a significant effect of days post-weaning on both ZO-1 (*p* = 0.004) and occludin expressions (*p* = 0.007), confirming that the decrease in tight junction protein expression is primarily driven by the weaning process. An independent effect of weaning age was observed for ZO-1 (*p* = 0.039), whereas no significant DPW × weaning age interactions were detected, suggesting that early post-weaning barrier gene modulation occurs largely irrespective of weaning age (Supplementary Materials S2).

The IAP and GLP-2 gene expressions in the duodenum were lower in the group weaned at 3 weeks of age as compared with later-weaned piglets (5-week group). Interestingly, weaning was associated with a transient increase in the duodenal IAP expression from 4 days post-weaning (3 weeks group, D22–D25: *p* = 0.028, D25–D32: *p* = 0.012), where suckling piglets showed a gradual decreasing trend in the IAP expression with age (5-week group, D22–D36, *p* = 0.037). The DPW analysis confirmed these observations, showing significant main effects for days post-weaning (*p* = 0.030), weaning age (*p* = 0.001), and a significant DPW × weaning age interaction (*p* = 0.002) for duodenal IAP. Early-weaned piglets had a higher duodenal IAP expression at 4 days post-weaning compared with piglets weaned at 5 weeks of age (*p* < 0.001). In contrast, the duodenal GLP-2 expression was not affected by the DPW or weaning age, suggesting minimal early post-weaning modulation.

In the mid-jejunum, the weaning effect is more clear, as expressions of both IAP and GLP-2 were significantly reduced in the weaned piglets (3 weeks group) as compared with age-matched suckling piglets (5 weeks group) (IAP expression, D25: *p* = 0.0007, D32: *p* = 0.001, D36: *p* = 0.0007) (GLP-2 expression, D25: *p* = 0.032, D32: *p* = 0.001, D36: *p* = 0.002). The DPW analysis supported these observations, with significant effects of DPW on both IAP (*p* = 0.017) and GLP-2 (*p* = 0.015). These findings indicate that early post-weaning changes in mid-jejunal IAP and GLP-2 expressions are primarily driven by the weaning event itself rather than the age at which the piglets were weaned.

### 3.4. Microbiota Analysis

#### 3.4.1. Microbial Diversity Is Influenced by Weaning of Piglets

In order to evaluate differences in the gut microbiota during early and late weaning, we analyzed the colonic content using 16S rRNA gene sequencing while implementing the same linear mixed-effects model. First, we examined alpha diversity by measuring the estimated species richness (Chao1 index) and the estimated community diversity (Shannon index).

**Figure 5:**
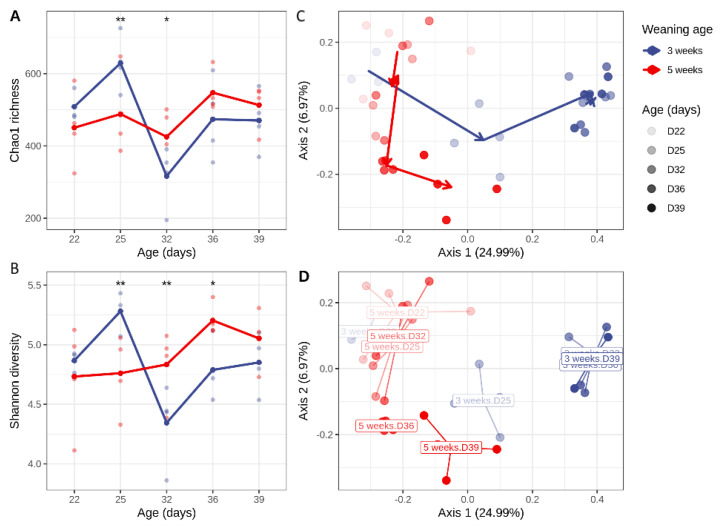
Effect of weaning on the alpha and beta diversities in the colonic microbiome. Alpha diversity is plotted as Chao1 richness (**A**) or Shannon index (**C**). PCoA plot of Bray–Curtis distances represented either as trajectories over time (**B**) or spider plots (**D**) to better visualize separate clustering of weaned piglets versus suckling piglets. Suckling piglets are clustered on the left, while weaned piglets cluster together on the right. Piglets at 4 days post-weaning (D25 of the group weaned at 3 weeks and D39 of the group weaned at 5 weeks) are in between the suckling and weaned piglets. Asterisks above timepoints represent the following levels of significance: “**” 0.01, “*” 0.05.

Weaning resulted in a significant increase of alpha diversity on D25 compared with age-matched suckling piglets (Chao1 *p* = 0.007; Shannon *p* = 0.005), followed by a sharp decline in alpha diversity on D32 (Chao1 *p* = 0.029; Shannon *p* = 0.007) before recovery at later timepoints. These changes could not be attributed to the age of the piglets, as no age effect was observed in the suckling animals (Figure 5A,B; Table S7).

When focusing the analysis of days post-weaning (DPW) and weaning age, a significant DPW × weaning age interaction for both Chao1 richness (*p* = 0.031) and Shannon diversity (*p* = 0.013) was observed. This indicates that the early post-weaning trajectory of microbial diversity differed depending on the weaning age. The main effects of DPW or weaning age alone were not significant for either diversity metric.

The pairwise comparisons showed that at 4 days post-weaning, the early-weaned piglets (3 weeks) had higher Chao1 richness than the later-weaned piglets (5 weeks) (629.0 ± 76.1 vs. 513.0 ± 65.5; *p* = 0.022), while the Shannon diversity showed a similar trend but did not reach statistical significance (5.28 ± 0.15 vs. 5.05 ± 0.24; *p* = 0.092). At 1 day post-weaning, the Shannon diversity was slightly higher in the 5-week group (5.20 ± 0.13 vs. 4.87 ± 0.09; *p* = 0.033), whereas the Chao1 richness did not differ significantly between the groups (Supplementary Materials S2).

The Bray–Curtis dissimilarity was used to compare the beta diversity between the two groups. Weaning and age accounted for, respectively, 13.9% (*p* = 0.001) and 17.5% (*p* = 0.001) of the variation in the piglet microbiota. Furthermore, a clear interaction between the age of the animals and the weaning group was observed, which accounted for 14.9% of the variation (*p* = 0.001). As long as the piglets were suckling, there was no difference in microbial community composition between animals from different ages (Table S9; 5-week group before weaning: D22–D32). After weaning, the beta diversity was significantly different from the suckling piglets (Table S9). This is also reflected on the beta diversity plots, where two clear clusters can be observed. The left cluster contains the samples from suckling piglets together with the samples from the early-weaned piglets at one day post-weaning (i.e., D22 samples of the 3-week group and D22 to D32 samples from the 5-week group). All samples from 10 days post-weaning onwards form a separate cluster with significantly different microbial beta diversity as compared with the suckling microbiome (Table S9). The samples from piglets at 4 days post-weaning (D25 of the 3-week group and D39 of the 5-week group) cluster together and form a transition between the suckling piglets and weaned piglet microbiomes (Figure 5C,D).

#### 3.4.2. Microbial Profile Is Influenced by Weaning

**Figure 6:**
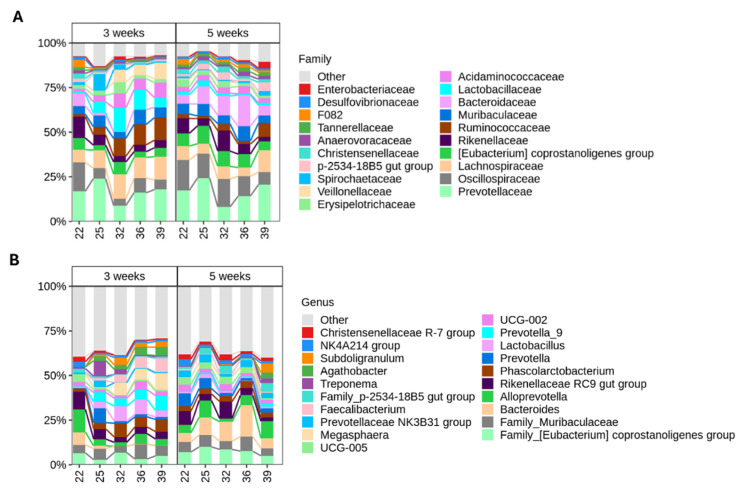
(**A**) Top 20 families. (**B**) Top 20 genera in piglets weaned at 3 weeks of age (**left side**) compared with suckling piglets weaned at 5 weeks of age (**right side**).

Weaning induces major shifts in the piglet gut microbiota. To visualize these changes, we plotted the top 20 most abundant families and genera from colonic content collected at each sampling timepoint (Figure 6A,B).

**Figure 7:**
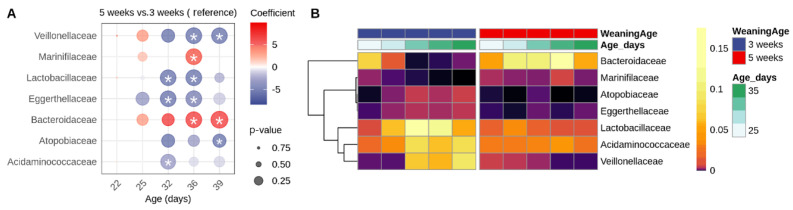
(**A**) Bubble plot showing differentially abundant families between piglets weaned at 5 weeks versus 3 weeks weaning age. The color scale represents the regression coefficient: red indicates higher abundance in the 5-week group, blue indicates higher abundance in the 3-week group. The circle size reflects the significance level (*p*-value), with larger circles indicating more significant differences. (**B**) Heatmap showing the relative abundance of significantly differentially abundant families. Asterisk represents significant differences.

**Figure 8:**
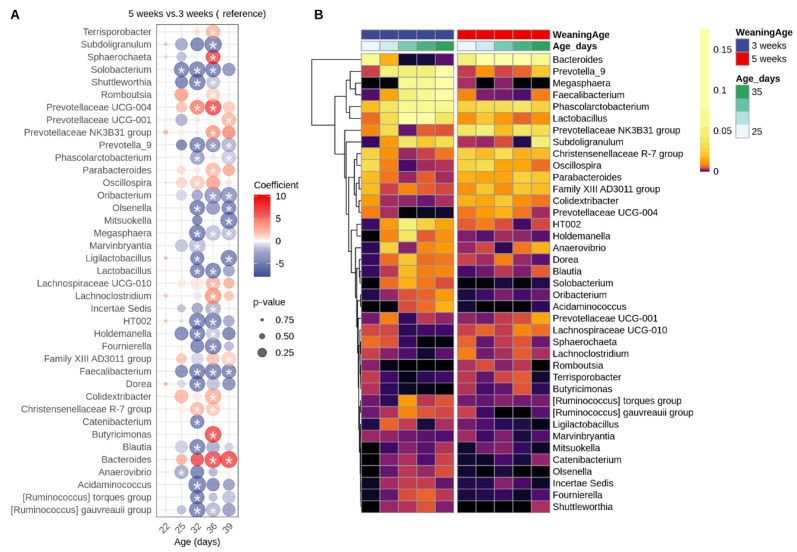
(**A**) Bubble plot showing differentially abundant genera between piglets weaned at 5 weeks versus 3 weeks weaning age. The color scale represents the regression coefficient: red indicates higher abundance in the 5-week group, blue indicates higher abundance in the 3-week group. The circle size reflects the significance level (*p*-value), with larger circles indicating more significant differences. (**B**) Heatmap showing the relative abundance of significantly differentially abundant genera. Asterisk represents significant differences.

To further investigate the differences in specific taxa, LinDA analysis was performed at the phylum, family and genus levels (Figure 7 and Figure 8). Weaning-induced microbial shifts were detectable as early as 4 days post-weaning (D25) and became the most pronounced from 10 days post-weaning onwards (D32), highlighting the gut microbiota’s rapid response to changes in the diet and environment. At the phylum level, the *Firmicutes*, *Actinobacteriota* and *Bacteroidota* levels were higher in early-weaned piglets at D39 (18 days post-weaning) compared with piglets weaned at 5 weeks of age, whereas the latter group showed increased *Campylobacterota* (Supplementary File S2). At the family level, the *Lactobacillaceae*, *Eggerthellaceae* and *Acidaminococcaceae* levels were elevated in the weaned piglets at D32 (11 days post-weaning) as compared with the age-matched suckling piglets. Additionally, at later timepoints, the early-weaned piglets showed a significant increase in *Veillonellaceae*, *Eggerthellaceae*, *Atopobiaceae* and *Acidaminococcaceae,* together with a decrease in *Marinifilaceae* and *Bacteroidaceae* relative to piglets weaned at 5 weeks of age (Supplementary File S2).

At the genus level, *Bacteroides* was highly abundant in suckling piglets and gradually increased over the suckling period, reaching 23% relative abundance in piglets weaned at 5 weeks. Early weaning at 3 weeks caused a rapid decline in *Bacteroides*, dropping from 10% at weaning to 1.8% at 4 days post-weaning and falling below 0.1% from 10 days post-weaning onwards. Weaning at 5 weeks led to a decrease in *Bacteroides* to a 7% relative abundance at 4 days post-weaning. Similar temporal patterns, though at lower overall abundance, were observed for *Prevotellaceae UCG-004* and *Lachnospiraceae UCG-010*. These shifts were accompanied by an enrichment of genera involved in fiber degradation, including *Prevotella_9*, *Megasphaera*, *Faecalibacterium, Subdoligranulum*, the *[Ruminococcus] gauvreauii group* and others. Additionally, *Lactobacillus*, *HT002* and the *[Ruminococcus] torques group* were significantly enriched after weaning, indicating a functional transition of the gut microbiota toward solid-feed fermentation and short-chain fatty acids production.

To distinguish age-dependent from weaning-specific microbiota dynamics, we analyzed samples at matched days post-weaning. Differential abundance testing showed that early weaning induced extensive community restructuring between DPW1 and DPW4 (22 genera significantly altered), while the late-weaned piglets exhibited minimal changes (only *Subdoligranulum* increased; Supplementary Materials S2). However, when comparing the early-weaned versus later-weaned piglets at different timepoints post-weaning, at 1-day post-weaning, the early-weaned piglets (3 weeks) had significantly lower levels of the genus *Rikenellaceae RC9 gut group* compared with the piglets weaned at 5 weeks (*p* = 0.018). At 4 days post-weaning, no differences between the piglets weaned at 3 versus 5 weeks were observed. These contrasting succession patterns suggest that early weaning destabilizes the microbiota, requiring extensive reorganization, whereas late weaning permits more resilient microbial communities during the immediate post-weaning transition.

## 4. Discussion

Establishing homeostasis is a prerequisite for a healthy gut with a robust microbial community. This balance is disrupted during weaning, with the earlier the ablactation, the more adverse the effects on the piglet’s health. Indeed, weaning coincides with the period where the GI barrier and the mucosal immune system is not yet fully developed [31,32], resulting in added pressure on the piglet’s already stressed state. For this reason, we aimed to investigate gut morphological changes in the small intestine, along with the associated gene expression and microbiota composition in the colons of piglets weaned at 3 and 5 weeks of age. Most studies that compare piglets before and after weaning compare post-weaning samples to samples collected at an earlier pre-weaning time point, which confounds the weaning event with normal biological maturation [33,34,35]. The present study included age-matched suckling controls up till 11 days post-weaning, allowing the effect of weaning to be isolated from developmental age effects. A linear mixed model was implemented to study the influence of age and weaning age of the piglet as well as their interaction, while taking into account the possible influence of the sow. However, it is worth noting that a drawback of this study design was the inability to include pen effects in the statistical model, as an entire pen was sampled at each timepoint. This means there was room for potential bias, as factors such as the environment and social dynamics could not be accounted for. In addition to the pen effect, a higher number of animals could have been used in order to better extrapolate the results to a wider population.

From D32 onwards, suckling piglets weighed more than those weaned at 3 weeks of age. Despite this difference in weight between the two groups, it has been shown that weaning age does not appear to impact the weight of same-aged pigs in the finisher stage. However, piglets weaned at a later age, i.e., 28 days seem to have a better adaptation during the post-weaning period, along with an improved removal rate in the nursery phase [36].

The small intestinal length-to-body weight ratio was higher in animals weaned at 3 weeks of age. In contrast with these results, it has been shown that early weaning in mice reduced the length of the small intestine in comparison with suckling pups [37]. The differences in intestinal length seen between the two groups in this study could be related to the type and frequency of feed intake, as has been reported elsewhere [38]. For example, diets rich in fibers, such as coarsely-ground oat hulls for pigs or crumbled instead of mashed grain-based diets for laying hens, affect the GIT by increasing its length and/or weight [39,40]. This led to the suggestion that a smaller length may indicate energy allocated to productivity rather than maintenance. This could be reflected in the weight difference observed between the two groups of this study.

Intestinal villi are fundamental in nutrient absorption and rely on crypts to provide new cells for their regeneration [41]. Weaning disrupts this balance, causing villus atrophy and enlarged crypt depth [4,42], as observed in the animals weaned at 3 weeks of age. Furthermore, immunohistochemistry staining of CD3^+^ T-cells, which are considered a marker for intestinal inflammation, were significantly higher 11, 15 and 18 days post-weaning at 3 weeks of age, indicating a prolonged inflamed status that was not seen in the other group.

In addition to their well-established role in regulating inflammation, cytokines also influence growth [43] and intestinal permeability [44]. Given that weaning is closely associated with these factors, we investigated the expressions of cytokines IL-8, FN-γ and TGF-β and saw changes in expression between age, groups and segments. IL-8 is a chemoattractant for polymorphonuclear cells (PMNs), such as neutrophils [45], and other pro-inflammatory cytokines that contribute to chronic inflammation and could potentially prolong and exacerbate the inflamed status seen right after weaning, while IFN-γ has a pleiotropic effect and is detected and enriched in the mucosa. It potentiates the effects of TNF-α, which is known to be involved in increased intestinal permeability [44]. Both cytokines have been previously shown to be affected by weaning [33]. This transient upregulation of IFN-γ expression induced by early weaning likely reflects localized immune activation and response to post-weaning changes in diet and microbiota. Finally, TGF-β is produced by immune and non-immune cells in the gut. It is known for modulating immune responses like T-cell activity and tissue remodeling [46], along with regulating the mucosal immune response. As an example, stromal TGF-β aids in preventing NF-κB from being translocated into the nucleus of monocytes; thereby reducing a pro-inflammatory response [47]. The differences in cytokine expression levels between intestinal segments have been previously reported and can be influenced by gut microbiota, weaning, diet or infection status [22,48,49,50,51].

pBD1 expression was higher and consistently increased in the duodenum of early-weaned piglets compared with the 5-week group. Defensins are host defense peptides produced in various epithelial cells and macrophages with antimicrobial and chemotactic properties [52]. Furthermore, cytokines produced by innate and adaptive immune cells can regulate how antimicrobial proteins respond to environmental stressors and pathogens [53]. For example, human beta defensin-1 (hBD-1)’s mRNA expression in activated mononuclear phagocytes is induced by IFN-γ or hBD-3 gene expression is promoted by IFN-γ in human primary keratinocytes [54,55]. Interestingly, human patients with IBD can have elevated levels of hBD-2, which could be an indication of a low-grade innate immune system activation despite the absence of macroscopic signs [56]. Similarly, some pigs may not show signs of inflammation but could potentially have elevated levels of pBD1. The change in diet and raised levels of IFN-γ in the 3-week weaned animals could explain the induced expression of this defensin.

The tight junction proteins ZO-1 and occludin expressions were decreased in the mid-jejunum of animals weaned at 3 weeks of age. Tight junctions are comprised of transmembrane and membrane-associated proteins that act as a seal between epithelial cells and block access of microorganisms and antigens to the basolateral side of the epithelium and ultimately entering the circulation, which would trigger the mucosal immune system [9]. Weaning is known to affect intestinal permeability [57], leading to a “leaky gut”. *In vitro*, upon binding to its receptor, IFN-γ induces paracellular permeability via rearrangement of actin and tight junction proteins by endocytosis of ZO-1 or occludin structures [44]. Furthermore, IFN-γ, in combination with TNF-α, has been shown to downregulate the transcription of occludin by suppressing its promoter [58], which could explain the low mRNA levels seen in the 3-week weaned piglets in the mid-jejunum.

In both the duodenum and mid-jejunum, lysozyme expression was transiently increased 11 days post-weaning in the 3-week group. Lysozyme is found throughout the body: in milk, saliva, liver, blood and mucosa, but also macrophages and neutrophils. More specifically in the gut, Paneth cells (PCs) [59] and Brunner’s glands [60] are reported as big producers of the enzyme. Apart from its antibacterial properties, lysozyme plays a role in modulating the innate immune response during infection [11], while its levels have been known to be elevated in chronic inflamed states, such as IBD, i.e., increased lysozyme levels in fecal [61] and colonic mucosa [62] samples. Contrary to our results, weaned mice had decreased lysozyme mRNA levels compared with suckling pups [37]. PCs existence in pigs has been widely contested; however, it is believed they exist, albeit different in structure and prevalence [63,64] when compared with humans or mice. In mice, PCs are a major source of lysozyme, and therefore, the impact weaning has on PC structure and numbers may lower lysozyme expression.

In this study, IAP expression was elevated in the mid-jejunum of suckling piglets. IAP is an important enzyme and a key marker when looking into primary digestive and absorptive function, as its expression is dependent on enterocyte differentiation [65]. Additionally, IAP prevents a TLR4-type inflammation reaction by dephosphorylating LPS [12] and/or dephosphorylating luminal ATP [66]. The expression and activity of IAP are known to be modulated by various factors. Consequently, the observed differences between the two groups may be attributed to the following variables: (1) Changes that occur in the small intestine due to weaning, e.g., acute villus atrophy. Since IAP is a brush border enzyme, atrophy can reduce IAP expression because less differentiated cells are present [67]. (2) Oxidative stress and its influence on caudal-associated homeobox transcription factor 1 (cdx1), which is a protein that activates the IAP gene [68]. Weaning results in oxidative stress, specifically ROS-induced stress [69], that silences cdx1 expression [70], as well as a decreased cdx1 protein abundance that has been shown to be associated with the reduced relative abundance of jejunal IAP mRNA [67]. Therefore, downregulation of cdx1 could explain the lower levels of IAP seen in the 3-week weaned piglets. (3) Diet can modulate the expression and/or IAP activity, e.g., carbohydrates such as lactose and cellulose stimulate activity, whereas fasting decreases IAP expression [71,72]. During weaning, there is a switch in diet, which can induce temporary anorexia. (4) Gut microbiota and short-chain fatty acids (SCFAs). Acetate and lactate can be used as substrates by butyrate-producing bacteria to produce butyrate, which is a known SCFA that increases IAP activity [12]. Therefore, remodeling of the gut bacteria and availability of substrates during the weaning phase can influence levels of SCFAs, and thus, IAP activity.

Regarding GLP-2, the main difference between the groups was seen in the mid-jejunum with the 5-week group, which had significantly higher expression levels on D32 and D36 compared with the early-weaned piglets. GLP-2 is present in low levels in the plasma but secretion from L-cells is increased upon sensing carbohydrates and lipids following feed ingestion [73]. More specifically, duodenal and jejunal L-cells are more nutrient-responsive than colonic L-cells, which mainly respond to secondary bile acids, SCFAs and microbial metabolites. These, in return, can stimulate and regulate the secretion of GLP-1, which unlike GLP-2, is involved in appetite, gastric emptying and insulin biosynthesis and secretion [15]. Because GLP-2 secretion is induced by nutrient intake, e.g., glucose and fat [74], it is possible that the difference seen between the two groups is due to the diet composition and frequency of eating. Together, these age-matched findings reveal distinct gene expression profiles between weaning ages, but the mechanistic basis for these differences (whether driven by the weaning event itself or by biological maturity at weaning) requires further analysis.

Our dual analytical approach, comparing piglets at both matched biological ages and matched days post-weaning, provides novel insights into the relative contributions of developmental maturity versus the weaning event itself. The DPW analysis revealed that acute morphological responses (increased crypt depth, immune cell infiltration) are remarkably consistent regardless of weaning age, suggesting these represent universal stress responses to dietary and housing transitions. However, the divergent adaptive patterns observed at 4 days post-weaning indicate that biological maturity fundamentally shapes recovery trajectories. The compensatory IAP upregulation in early-weaned piglets is particularly noteworthy. IAP plays critical roles in detoxifying lipopolysaccharide, maintaining gut barrier function, and regulating microbiota composition. The 4-fold elevation in duodenal IAP expression at 4 days post-weaning in the 3-week group as compared with the 5-week group may represent an adaptive mechanism to cope with increased microbial antigen exposure in less mature intestinal tissues. Conversely, the sustained elevation of IL-8 and lysozyme in the 5-week group at 4 days post-weaning suggests that more mature piglets maintain robust antimicrobial defenses without requiring compensatory upregulation. The contrasting microbiota dynamics further support an age-dependent adaptive model. Early weaning induced dramatic microbiota restructuring, with 22 genera significantly altered between one and four days post-weaning, indicating a less stable microbial community requiring substantial reorganization. Late-weaned piglets exhibited minimal compositional changes, suggesting a more resilient microbiota capable of withstanding weaning stress. This stability may reflect a more mature and diverse pre-weaning microbiota, as evidenced by the higher Shannon diversity at one day post-weaning in the 5-week group. These findings reconcile previous contradictory reports in the literature by clarifying that weaning age does not prevent the acute stress response but modulates the adaptive capacity and recovery kinetics. Producers may benefit from the extra nursing days not through avoidance of weaning stress, but through enhanced resilience and more stable post-weaning trajectories.

This pattern of universal stress responses paired with age-dependent adaptive capacity extends to the microbiota. The alpha diversity of piglets weaned at 3 weeks of age increased 4 days post-weaning, followed by a sudden drop 11 days post-weaning before gradually recovering. The alpha diversity of suckling piglets appeared more stable. Alpha diversity has been reported to both increase [75,76] and decrease [77,78] after weaning; however, it eventually stabilizes as the pig matures [79]. Beta diversity was influenced by both weaning and age, with suckling piglets clustering separately from the early-weaned piglets, while those that were sampled 4 days post-weaning from either group clustered together. This indicates a transitory period from one developmental stage to the next. Apart from the role diet has in shaping the microbiota, age is an important contributing factor as the microbial community becomes more homogenous with age [80,81].

Diet is known to shape the gut microbiota [82], with piglets gradually shifting from a population that contains bacteria processing simple and easily digestible carbohydrates derived from sow’s milk to having higher abundant taxa that can break down more complex plant-derived carbohydrates. The differences seen in diversity reflects the switch in diets, as do the genera that colonize the gut. Specifically, piglets belonging to the 3 weeks group had significantly higher levels of *Prevotella_9*, *Megasphaera*, *Faecalibacterium* and *Subdoligranulum* 11 days after weaning, as has been reported elsewhere [83]. These taxa are able to metabolize smaller plant monosaccharides or use end products from other bacteria, e.g., lactate to produce SCFAs [2,84,85]. Moreover, they have been associated with piglets with high average daily gain [86], while *Faecalibacterium* was linked to healthy weaned piglets [87]. In addition, a higher abundance of *Prevotella* has been linked to improved growth performance and better protection against diarrhea [88]. Consistent with previous reports [86,89,90], *Bacteroides*, which utilize a variety of saccharides and efficiently uses the milk-derived carbohydrates [90,91], were prevalent in suckling piglets but reduced in early-weaned piglets, which reflects the loss of milk-derived substrates. Their elevated levels persisted 4 days post-weaning at 5 weeks of age, with the same trend being observed post-weaning elsewhere [83]. Finally, *Lactobacillus* is considered to be predominant in suckling piglets but decreases after weaning [76,92]; however, its relative abundance was higher in the 3 weeks group 11 days after weaning compared with suckling piglets. A similar increase in *Lactobacillus* after weaning has been observed [89]. An explanation for this could be that some *Lactobacilli* encode carbohydrate-utilizing genes and are capable of metabolizing starch [93,94], therefore taking advantage of a nutrient-rich environment.

Given these results, it is evident there is an interplay of multiple factors such as diet change, stress and inflammation brought forth by weaning, which are key contributors to the piglet’s welfare. For example, stress and inflammation caused by reduced or altered feed intake during the first days after weaning leads to oxidative stress, resulting in the production of compounds such as nitric oxide (NO) [2,95]. In the intestinal lumen, NO is converted into nitrate, which promotes a beneficial environment for bacteria such as ETEC that encode nitrate reductase, hence potentiating a shift towards dysbiosis [1,2,92]. Furthermore, suckling piglets that remain on sow’s milk ingest higher amounts of glutamine and glutamate that have been shown to be the most abundant amino acids throughout lactation [96], which can benefit the host as glutamine is used as fuel by immune cells and aids in intestinal development [97,98].

## 5. Conclusions

To summarize, our results showed that early weaning induces changes to gut morphology, as well as gene expression involved in inflammation, function and maintenance of homeostasis. Furthermore, weaning was responsible for alpha diversity to fluctuate in piglets weaned at 3 weeks of age, while an interaction between weaning and age contributed to the differences seen in beta diversity. Finally, weaning affected the taxa at the family and genus levels, with a shift from milk-substrate- to complex-carbohydrate-utilizing bacteria.

## Figures and Tables

**Table 1:** Ingredient and chemical composition of post-weaning diet.

Ingredients	%	Description	Value	Unit
Barley	26	Dry matter	895.3	g/kg
Wheat	25	Humidity	104.7	g/kg
Toasted Soybeans	13	Crude protein (excl NH_3_)	175	g/kg
Corn	10	Crude fat	49.8	g/kg
Premix weaner ^1^	9	Crude ash	53.6	g/kg
Soybean meal	6	Crude fiber	34.1	g/kg
Beet molasses	3	Nev	10	MJ/kg
Potato protein	2	Starch Ewers	354.5	g/kg
Wheat gluten	1.571	Sugars	97.8	g/kg
Monocalciumphosphate	0.923	Lactose	55.7	g/kg
Limestone	0.747	NSP	148.9	g/kg
L-Lysine HCL	0.604	ADF	39	g/kg
Salt	0.492	ADL	6.2	g/kg
L-threonine	0.259	NDF	99.1	g/kg
DL-methionine	0.243	Sodium	2.5	g/kg
L-Valine	0.160	Potassium	9.7	g/kg
L-Tryptophan	0.079	Chlorine	6.7	g/kg
Phytase (1000 FYT/g)	0.1	EB	167.8	meq/kg
L-Leucine	0.044	Calcium	5.9	g/kg
L-Isoleucine	0.017	Phosphorus	5.5	g/kg
		P digestible Swine	4.2	g/kg
		Ca/P	1.093	
		Ca/P digestible	1.417	

^1^ Premix composition can be found in Supplementary Materials S3.

**Table 2:** qPCR primer sequence for GOI and HKGs.

Gene	Sequence	Fragment Size (bp)	Reference
*B2M*	F: 5′-CCCCGAAGGTTCAGGTTTAC-3′	229 bp	Martin et al. 2013 [20]
	R: 5′-CGGCAGCTATACTGATCCAC-3′		
*RPL19*	F: 5′-GCTTGCCTCCAGTGTCCTC-3′	78 bp	Martin et al. 2013 [20]
	R: 5′-GCGTTGGCGATTTCATTAG-3′		
*RPL4*	F: 5′-GAGAAACCGTCGCCGAAT-3′	146 bp	Loos et al. 2012 [21]
	R: 5′-GCCCACCAGGAGCAAGTT-3′		
*Cyclophilin*	F: 5′-TGCTTTCACAGAATAATTCCAGGATTTA-3′	77 bp	de Groot et al. 2021 [22]
	R: 5′-GACTTGCCACCAGTGCCATTA-3′		
*GAPDH*	F: 5′-ACATGGCCTCCAAGGAGTAAGA-3′	106 bp	de Groot et al. 2021 [22]
	R: 5′-GATCGAGTTGGGGCTGTGACT-3′		
*IL-8*	F: 5′-GCTCTCTGTGAGGCTGCAGTTC-3′	79 bp	Qi et al. 2021 [23]
	R: 5′-AAGGTGTGGAATGCGTATTTATGC-3′		
*IFN-γ*	F: 5′-TTCAGCTTTGCGTGACTTTG-3′	121 bp	Qi et al. 2021 [23]
	R: 5′-GGTCCACCATTAGGTACATCTG-3′		
*TGF-β*	F: 5′-CACGTGGAGCTATACCAGAA-3′	108 bp	de Groot et al. 2021 [22]
	R: 5′-TCCGGTGACATCAAAGGACA-3′		
*pBD1*	F: 5′-TAACCTGCTTACGGGTCTTG-3′	167 bp	Zhang et al. 2023 [24]
	R: 5′-TGCTGTGGCTTCTGGCTC-3′		
*IAP*	F: 5′-AAGGGGCAGATGAATGGCAA-3′	165 bp	Martin et al. 2013 [20]
	R: 5′-CATATGGGTCTTGACCCCGC-3′		
*GLP-2*	F: 5′-ACTCACAGGGCACGTTTACCA-3′	149 bp	Zhou et al. 2021 [25]
	R: 5′-AGGTCCCTTCAGCATGTCTCT-3′		
*Lysozyme*	F: 5′-AATAGCCGCTACTGGTGTAATGATG-3′	148 bp	Wang et al. 2020 [26]
	R: 5′-ATGCTTTAACGCCTAGTGGATCTCT-3′		
*ZO-1*	F: 5′-CCTGAGTTTGATAGTGGCGTTGA-3′	269 bp	Qi et al. 2021 [23]
	R: 5′-AAATAGATTTCCTGCTCAATTCC-3′		
*Occludin*	F: 5′-ATCAACAAAGGCAACTCT-3′	157 bp	Xia et al. 2017 [27]
	R: 5′-GCAGCAGCCATGTACTCT-3′		

*B2M*: beta-2 microglobulin; *RPL19*: ribosomal protein L19; *RPL4*: ribosomal protein L4; *GAPDH*: glyceraldehyde-3-phosphate dehydrogenase; *IL-8*: interleukin 8; *IFN-γ*: interferon-gamma; *TGF-β*: transforming growth factor-beta; *pBD1*: porcine beta defensin 1; *IAP*: intestinal alkaline phosphatase; *GLP-2*: glucagon-like peptide 2; *ZO-1*: zonula occludens-1.

## Data Availability

The sequencing data are available on NCBI SRA under the BioProject PRJNA1357625. All other original contributions presented in this study are included in the article, and the raw data supporting the conclusions of this article will be made available by the authors upon request.

## Supplementary Materials

The following supporting information can be downloaded at: https://www.mdpi.com/article/10.3390/ani16060961/s1, Supplementary File S1: Table S1: Effect of weaning on intestinal morphology and CD3^+^ T-cell abundance in the mid-jejunum; Table S2: ANOVA and pairwise post hoc results of linear mixed-effects model for villus length in the mid-jejunum; Table S3: ANOVA and pairwise post hoc results of linear mixed-effect model for crypt depth in the mid-jejunum; Table S4: ANOVA and pairwise post hoc results of linear mixed-effect model for CD3 area percentage in the mid-jejunum; Table S5: ANOVA and pairwise post hoc results of linear mixed-effects model for qPCRs of genes of interest in duodenum; Table S6: ANOVA and pairwise post hoc results of linear mixed-effects model for qPCRs genes of interest in mid-jejunum; Table S7: Linear mixed model with SowID & Age included as a random effect for alpha diversity; Table S8: Bray–Curtis: multivariate homogeneity of groups dispersions (variances)—betadisper; Supplementary File S2: LinDA results; Supplementary Materials S1: Table S9: PERMANOVA and Wd*-test *p*-values for weaning age and age effects on the beta diversity between the two groups; Supplementary Materials S2: The effect of weaning age on epithelial morphology, gene expression and gut microbiota composition in piglets; Supplementary Materials S3: Premix weaner.

## Abbreviations

The following abbreviations are used in this manuscript:

ETEC	Enterotoxigenic *Escherichia coli*
PWD	Post-weaning diarrhea
ZO-1	Zonula occludens-1
pBD1	Porcine beta-defensin-1
IAP	Intestinal alkaline phosphatase
GLP-2	Glucagon-like peptide 2
GIT	Gastrointestinal tract
PBS	Phosphate-buffered saline
CTAB	Cetyltrimethylammonium bromide
hBD-1	Human beta defensin-1
PC	Panth cell
cdx1	Caudal-associated homeobox transcription factor 1
SCFA	Short-chain fatty acids
NO	Nitric oxide
DPW	Days post-weaning
