# Supplementary material for: Weaning Influences Epithelial Morphology, Gene Expression and Gut Microbiota Composition in Piglets
Source: Animals (Basel). 2026 Mar 19;16(6):961. doi: 10.3390/ani16060961 (PMC13023247; doi:10.3390/ani16060961)
Supplement: Supplementary file 1 [file animals-16-00961-s001.zip › Supplementary Materials S1.pdf]

## Supplementary Materials

**Table S9.** PERMANOVA and Wd*-test p values for Weaning Age and Age effect on the beta diversity between the two groups. Wd*test was performed as it does not require equal group variance since the assumption of equal dispersion was not met for D39. Both the p and adjusted p values of the Age and Weaning Age effects from the PERMANOVA and Wd*test are included.

| | **Weaning Age** | | |
|---|---|---|---|
| 5 weeks vs 3 weeks | PERMANOVA | Wd*test | |
| Days | P value | P value | P value adj |
| 22 | 0.857 | 0.841 | 0.841 |
| 25 | 0.027 | 0.026 | 0.047 |
| 32 | 0.028 | 0.029 | 0.047 |
| 36 | 0.027 | 0.031 | 0.047 |
| 39 | 0.025 | 0.019 | 0.047 |

| | **Age** | | | |
|---|---|---|---|---|
| 3 weeks | PERMANOVA | | Wd*test | |
| Days | P value | P value adj | P value | P value adj |
| 25vs22 | 0.028 | 0.045 | 0.025 | 0.047 |
| 32vs22 | 0.028 | 0.045 | 0.031 | 0.047 |
| 36vs22 | 0.028 | 0.045 | 0.034 | 0.047 |
| 39vs22 | 0.028 | 0.045 | 0.027 | 0.047 |
| 32vs25 | 0.031 | 0.045 | 0.025 | 0.047 |
| 36vs25 | 0.030 | 0.045 | 0.03 | 0.047 |
| 39vs25 | 0.028 | 0.045 | 0.027 | 0.047 |
| 36vs32 | 0.285 | 0.316 | 0.293 | 0.321 |
| 39vs32 | 0.115 | 0.144 | 0.102 | 0.124 |
| 39vs36 | 0.568 | 0.568 | 0.554 | 0.593 |
| 5 weeks | | | | |
| 25vs22 | 0.659 | 0.685 | 0.664 | 0.693 |
| 32vs22 | 0.685 | 0.685 | 0.678 | 0.693 |
| 36vs22 | 0.028 | 0.049 | 0.042 | 0.054 |
| 39vs22 | 0.027 | 0.049 | 0.039 | 0.051 |
| 32vs25 | 0.118 | 0.148 | 0.114 | 0.135 |
| 36vs25 | 0.029 | 0.049 | 0.026 | 0.047 |
| 39vs25 | 0.028 | 0.049 | 0.029 | 0.047 |
| 36vs32 | 0.083 | 0.119 | 0.08 | 0.1 |
| 39vs32 | 0.027 | 0.049 | 0.028 | 0.047 |
| 39vs36 | 0.028 | 0.049 | 0.03 | 0.047 |
