# Supplementary material for: Weaning Influences Epithelial Morphology, Gene Expression and Gut Microbiota Composition in Piglets
Source: Animals (Basel). 2026 Mar 19;16(6):961. doi: 10.3390/ani16060961 (PMC13023247; doi:10.3390/ani16060961)
Supplement: Supplementary file 1 [file animals-16-00961-s001.zip › Supplementary Materials S2.pdf]

Supplementary Materials

The effect of weaning age on epithelial morphology, gene expression and gut microbiota composition in piglets

Sample selection

To further investigate the influence of weaning age on piglet physiology and associated parameters, we conducted an additional analysis focusing on days post-weaning for animals weaned at 3 or 5 weeks of age. Samples collected at two critical time point (1 and 4 days post weaning) were selected to capture early post-weaning dynamics. This supplementary analysis complements the main study by providing a more detailed view of how the timing of weaning affects the measured outcomes during the immediate post-weaning period.

Statistical analysis

All statistical analyses were performed in R (v4.2.1). For histological parameters and gene expression data, linear mixed-effects models were fitted using the following formula:

$$Response \sim DPW \; x \; WeaningAge + (1 \mid SowID)$$

where DPW represents days post weaning (1 or 4 days), WeaningAge indicates the weaning group (3 weeks vs 5 weeks), and SowID was included as a random intercept to account for sow-level variability. Post-hoc pairwise comparison between weaning groups at each DPW were performed using the *emmeans* package. P-values were adjusted for multiple comparison using Tukey's method. Although many interactions did not reach significance, simple effect contrasts at each DPW level are presented for descriptive purposes, as the study was underpowered to detect a differential response.

For microbiota analysis, differential abundance testing at phylum, family, and genus levels was performed using LinDA (linear models for Differential Abundance analysis) from the *MicrobiomeStat* package. LinDA accounts for the compositional nature of microbiome data and provides robust inference while controlling for false discovery rate.

Effect of weaning age on intestinal morphology of the mid jejunum

Linear mixed-effects model results showing the effect of days post-weaning (DPW), weaning age, and their interaction on villus length, crypt depth, and CD3+ T-cell area percentage in the mid jejunum. Values are presented as mean ± SD. Statistical comparisons between weaning groups (3 weeks vs. 5 weeks) at each DPW are shown with adjusted p-values from pairwise comparisons.

Type III Analysis of Variance

Effect	p-value	Significance
	**Crypt depth**	
DPW	0.074	ns
WeaningAge	0.957	ns
DPW x WeaningAge	0.452	ns
	**Villus length**	
DPW	0.352	ns
WeaningAge	0.374	ns
DPW x WeaningAge	0.866	ns
	**CD3 area percentage**	
DPW	**0.005**	**
WeaningAge	0.180	ns
DPW x WeaningAge	0.423	ns

Descriptive statistics and Pairwise Comparisons

DPW	3 weeks (mean ± SD)	5 weeks (mean ± SD)	p-value	Significance
		**Crypt depth**		
1	144.418 ± 27.810	134.958 ± 7.774	0.617	ns
4	160.351 ± 40.930	171.242 ± 12.889	0.566	ns
		**Villus length**		
1	340.342 ± 113.702	310.105 ± 63.046	0.603	ns
4	308.300 ± 48.212	264.228 ± 77.577	0.453	ns
		**CD3 area percentage**		
1	6.775 ± 1.427	7.150 ± 1.320	0.674	ns
4	4.000 ± 0.735	5.400 ± 1.606	0.140	ns

Note: DPW = Days Post-Weaning. Significance codes: *** $p < 0.001$, ** $p < 0.01$, * $p < 0.05$, ns = not significant.

Effect of weaning age on gene expression in duodenum and mid jejunum

Type III ANOVA results and descriptive statistics for relative gene expression of inflammatory markers (IL-8, INF-γ, TGF-β), tight junction proteins (ZO-1, Occludin), antimicrobial peptide (pBD1, Lysozyme), and functional markers (IAP, GLP-2) in duodenum and mid jejunum. Gene expression was analyzed using linear mixed-effects models with DPW, weaning age, and their interaction as fixed effects and SowID as random effect. Values are represented as mean ± SD of relative expression (normalized to reference genes).

Duodenum - Type III Analysis of Variance

Effect	p-value	Significance
	**IL-8**	
DPW	**0.021**	*
WeaningAge	0.125	ns
DPW x WeaningAge	0.089	ns
	**INF-γ**	
DPW	0.410	ns
WeaningAge	0.724	ns
DPW x WeaningAge	0.205	ns
	**TGF-β**	
DPW	0.311	ns
WeaningAge	0.622	ns
DPW x WeaningAge	0.639	ns
	**ZO-1**	
DPW	0.985	ns
WeaningAge	0.701	ns
DPW x WeaningAge	0.131	ns
	**Occludin**	
DPW	0.607	ns
WeaningAge	0.579	ns
DPW x WeaningAge	0.563	ns
	**pBD1**	
DPW	0.660	ns
WeaningAge	0.999	ns
DPW x WeaningAge	0.165	ns
	**Lysozyme**	
DPW	**0.046**	*
WeaningAge	0.068	ns
DPW x WeaningAge	0.135	ns
	**IAP**	
DPW	**0.030**	*
WeaningAge	**0.001**	**
DPW x WeaningAge	**0.002**	**
	**GLP-2**	
DPW	0.864	ns
WeaningAge	0.132	ns
DPW x WeaningAge	0.302	ns

Duodenum - Descriptive statistics and Pairwise Comparisons of weaning age effect at different days post-weaning

DPW	3 weeks (mean ± SD)	5 weeks (mean ± SD)	p-value	Significance
		**IL-8**		
1	0.521 ± 0.181	0.471 ± 0.046	0.882	ns
4	0.728 ± 0.366	1.567 ± 0.836	**0.031**	*
		**INF-γ**		
1	3.547 ± 6.411	1.008 ± 0.731	0.252	ns
4	0.275 ± 0.299	1.745 ± 1.218	0.496	ns
		**TGF-β**		
1	0.781 ± 0.086	0.775 ± 0.254	0.986	ns
4	1.150 ± 0.736	0.914 ± 0.533	0.500	ns
		**ZO-1**		
1	0.869 ± 0.326	1.092 ± 0.283	0.394	ns
4	1.165 ± 0.481	0.802 ± 0.282	0.180	ns
		**Occludin**		
1	0.915 ± 0.381	1.090 ± 0.319	0.427	ns
4	0.926 ± 0.296	0.922 ± 0.156	0.986	ns
		**pBD1**		
1	1.784 ± 2.517	0.655 ± 0.219	0.313	ns
4	0.994 ± 0.862	2.124 ± 1.341	0.313	ns
		**Lysozyme**		
1	0.512 ± 0.297	0.642 ± 0.460	0.766	ns
4	0.713 ± 0.502	1.824 ± 0.935	**0.027**	*
		**IAP**		
1	0.953 ± 0.375	0.823 ± 0.443	0.624	ns
4	2.203 ± 0.549	0.506 ± 0.234	**< 0.001**	***
		**GLP2**		
1	1.062 ± 0.337	1.257 ± 0.525	0.700	ns
4	0.743 ± 0.295	1.698 ± 1.239	0.083	ns

Mid jejunum - Type III Analysis of Variance

Effect	p-value	Significance
	**IL-8**	
DPW	0.954	ns
WeaningAge	0.547	ns
DPW x WeaningAge	0.248	ns
	**INF-γ**	
DPW	0.163	ns
WeaningAge	0.849	ns
DPW x WeaningAge	0.430	ns
	**TGF-β**	
DPW	**0.003**	**
WeaningAge	0.236	ns
DPW x WeaningAge	0.855	ns
	**ZO-1**	
DPW	**0.004**	**
WeaningAge	**0.039**	*
DPW x WeaningAge	0.186	ns
	**Occludin**	
DPW	**0.007**	**
WeaningAge	0.108	ns
DPW x WeaningAge	0.179	ns
	**pBD1**	
DPW	0.067	ns
WeaningAge	0.616	ns
DPW x WeaningAge	0.315	ns
	**Lysozyme**	
DPW	0.905	ns
WeaningAge	0.307	ns
DPW x WeaningAge	0.798	ns
	**IAP**	
DPW	**0.017**	*
WeaningAge	0.149	ns
DPW x WeaningAge	0.500	ns
	**GLP-2**	
DPW	**0.015**	*
WeaningAge	0.812	ns
DPW x WeaningAge	0.699	ns

Mid jejunum - Descriptive statistics and Pairwise Comparisons of weaning age effect at different days post-weaning

DPW	3 weeks (mean ± SD)	5 weeks (mean ± SD)	p-value	Significance
		**IL-8**		
1	1.059 ± 0.244	1.357 ± 1.058	0.680	ns
4	1.643 ± 1.279	0.753 ± 0.212	0.223	ns
		**INF-γ**		
1	2.324 ± 3.569	1.390 ± 0.552	0.490	ns
4	0.185 ± 0.061	0.796 ± 0.688	0.669	ns
		**TGF-β**		
1	2.182 ± 1.053	1.726 ± 0.498	0.326	ns
4	0.844 ± 0.182	0.529 ± 0.129	0.456	ns
		**ZO-1**		
1	2.842 ± 1.319	1.463 ± 0.220	**0.025**	*
4	0.909 ± 0.233	0.543 ± 0.286	0.483	ns
		**Occludin**		
1	2.937 ± 1.408	1.578 ± 0.691	**0.049**	*
4	0.824 ± 0.124	0.679 ± 0.066	0.810	ns
		**pBD1**		
1	1.006 ± 0.280	1.474 ± 0.676	0.294	ns
4	0.823 ± 0.715	0.802 ± 0.318	0.715	ns
		**Lysozyme**		
1	1.251 ± 0.563	1.697 ± 1.179	0.571	ns
4	1.045 ± 0.837	1.771 ± 1.043	0.364	ns
		**IAP**		
1	2.594 ± 0.492	2.163 ± 1.324	0.541	ns
4	1.481 ± 0.686	0.380 ± 0.251	0.142	ns
		**GLP2**		
1	2.629 ± 0.530	3.096 ± 2.271	0.660	ns
4	0.661 ± 0.462	0.552 ± 0.241	0.918	ns

Effect of weaning age on colon microbiota diversity and composition

Type III ANOVA results and descriptive statistics the alpha diversity calculated as the estimated species richness (Chao1 index) or the estimated community diversity (Shannon index). Alpha diversity was analyzed using linear mixed-effects models with DPW, weaning age, and their interaction as fixed effects and SowID as random effect. Values are represented as mean ± SD.

Type III Analysis of Variance

Effect	p-value	Significance
	**Chao1 richness**	
DPW	0.180	ns
WeaningAge	0.260	ns
DPW x WeaningAge	**0.031**	*
	**Shannon diversity**	
DPW	0.176	ns
WeaningAge	0.560	ns
DPW x WeaningAge	**0.013**	*

Descriptive statistics and Pairwise Comparisons

DPW	3 weeks (mean ± SD)	5 weeks (mean ± SD)	p-value	Significance
		**Chao1 richness**		
1	508.653 ± 44.810	547.280 ± 57.460	0.380	ns
4	629.010 ± 76.096	513.002 ± 65.515	**0.022**	*
		**Shannon diversity**		
1	4.867 ± 0.090	5.204 ± 0.133	**0.033**	*
4	5.281 ± 0.153	5.053 ± 0.240	0.092	ns

LinDA analysis results for colonic microbiota at phylum, family and genus levels. The analysis compared piglets weaned at 3 weeks versus 5 weeks at DPW 1 and DPW 4. No significant results were observed at either phylum or family level. At one day post-weaning, piglets weaned at 3 weeks had significantly lower levels of the genus *Rikenellaceae RC9 gut group* than piglets weaned at 5 weeks (p = 0.018). No other effects of weaning age on the microbiota composition were detected.
